# *In vitro* fermentation potential of diet-derived fermentable proteins of thirty-one human foods

**DOI:** 10.1017/S0007114525103541

**Published:** 2025-06-14

**Authors:** Hanlu Zhang, John W. Cone, Arie K. Kies, Wouter H. Hendriks, Nikkie van der Wielen

**Affiliations:** 1 Animal Nutrition Group, Department of Animal Sciences, Wageningen University & Research, Wageningen, The Netherlands; 2 State Key Laboratory of Animal Nutrition, College of Animal Science and Technology, China Agricultural University, Beijing, People’s Republic of China; 3 ArieKiesAdvies, Druten, The Netherlands; 4 Division of Human Nutrition and Health, Department of Agrotechnology and Food Sciences, Wageningen University & Research, Wageningen, The Netherlands

**Keywords:** Undigested protein, *In vitro* fermentation, Human inoculum, Porcine model, Gas production

## Abstract

Protein fermentation in the human gut is often associated with adverse health effects. Hence, understanding the fermentation characteristics of dietary undigested proteins is important for a comprehensive nutritional value of foods. This study investigated the protein fermentation kinetics of diet-derived proteins from thirty-one different foods using an *in vitro* model and human faecal inoculum. The undigested diet-derived protein substrate originated from porcine ileal digesta obtained from assessment of the digestible indispensable amino acid score (DIAAS) of the foods. Significant variations in fermentation kinetic parameters, particularly in maximum gas production rate (*R*_max_) and time to reach cumulative gas production (GP) from the substrate (T_GPs_), were observed. The R_max_ ranged from 15·5 (se 0·7) ml/h for wheat bran-derived to 24·5 (se 0·9) ml/h for oatmeal-derived proteins. Egg-derived proteins had the shortest T_GPs_ (14·7 (se 0·7) h), while mushroom-derived proteins had the longest (27·6 (se 7·1) h). When foods were categorised into five groups (‘animal protein’, ‘grains’, ‘legumes’, ‘fungi, algae and microorganisms’ and ‘others’), no significant differences were found in fermentation kinetics parameters. Samples were additionally incubated with porcine inoculum to assess potential donor-species effects. Human inoculum showed significantly lower R_max_, cumulative GP and microbiota turnover than porcine inoculum, indicating reduced fermentative activity. Linear regression analysis revealed correlations between human and porcine-derived inoculum only for R_max_ (R^2^ = 0·78, *P* < 0·01) and T_GPs_ (R² = 0·17, *P* < 0·05). These findings underscore the importance of using human inoculum in *in vitro* studies to better predict health implications of foods with DIAAS values.

Detrimental health effects associated with high-protein diets have been reported in humans and include impaired gut health caused by metabolites produced during protein fermentation by the gut microbiota^(1,2)^. The gut microbiota synthesises a vast array of proteases with serine (39·2 %), metallo- (35·9 %) and cysteine (16·5 %) proteases being the most abundant^(3)^. These proteases hydrolyse proteins into small peptides and amino acids (AA) which, after absorption, can yield various metabolites including short-chain fatty acids, ammonium, phenols, indoles and amines, in addition to gases such as CO_2_, H_2_ and H_2_S^(4)^. Many of these metabolites, as well as bacterial proteases, have physiological activity and can be toxicologically significant depending on their concentration^(3,5)^.

Besides undigested dietary proteins, endogenous proteins like mucin, which can be influenced by dietary protein source^(6)^, also enter the large intestine and undergo fermentation. Notably, microbial protein, though considered ‘non-dietary’, significantly contributes to the proteinaceous material in the digesta and is often included in endogenous protein measurement^(7)^. The fermentability of undigested dietary and endogenous proteins can be studied in greater detail using an *in vitro* gas production (GP) method utilising a nitrogen (N)-limiting environment^(8,9)^ and microbiota-containing inoculum, allowing for the large-scale screening of foods. The mathematical modelling of the cumulative GP in this method provides various parameters that describe the fermentation kinetics of the N-containing components by the microbiota^(8–13)^ and allows comparison of protein sources^(8)^. By utilising ileal digesta as a substrate rather than the consumed foods, in combination with human faecal inoculum, insights into the fermentation potential of undigested N-containing components that normally enter the large intestine of humans can be obtained. A recent study showed that true ileal digestibility values in growing pigs can be used directly to predict protein and AA digestibility in humans, indicating that ileal digesta corrected for endogenous losses are comparable between humans and pigs^(14)^. Pigs, due to their high anatomical and physiological similarity to humans, are increasing in value as a model to investigate relevant human digestive diseases^(15)^. As such the growing pig is nowadays used as a model for humans in relation to food digestion^(16)^, including for calculating the digestible indispensable amino acid score (DIAAS) recommended by the FAO^(17)^. Currently, the application of pigs for evaluating fermentation processes in the human digestive tract is relatively underexplored. Although some studies^(18–22)^ have utilised porcine ileal digesta to investigate the *in vitro* fermentation of fibre, data on protein fermentation are limited, and the suitability of porcine rather than human inoculum in these *in vitro* assays has not been validated.

In this study, we investigated the fermentation potential of undigested protein from thirty-one human foods including animal-based, grains, legumes, fungi, algae and microorganisms, and other proteins sources, such as isolates, using digesta collected from the terminal ileum of pigs. Digesta samples were part of DIAAS determination of the foods and were incubated using human faecal inoculum, alongside porcine inoculum to determine whether human inoculum is essential for accurately assessing the protein fermentation potential of human foods when determining a DIAAS value according to the FAO protocol.

## Material and methods

### Ethics

The collection of human faeces did not require an ethical approval, as declared by the Medical Ethics Committee of East Netherlands. The individuals who provided a faecal sample for *in vitro* fermentation were anonymised while their weight, height, age and sex were registered. Details regarding the ethics approval for the pig cannulation trial were published in the corresponding study^(14)^. The porcine faeces used for inoculum was the frozen sample as described in our previous study^(9)^.

### Ileal digesta

Ileal digesta samples were obtained from previous studies conducted to determine DIAAS values of foods according to the FAO (2013) protocol^(23)^. Detailed methods as employed are described by Hodgkinson *et al.* (2022) and Huang *et al.* (2023)^(14,24)^. The preparation of test foods and procedures for the *in vivo* study are described in the online Supplementary Material. Briefly, thirty-one different human foods were prepared in the same manner as they are consumed by humans and fed to ileal-cannulated growing pigs. The final diets contained 100 g crude protein per kg DM with titanium dioxide (TiO_2_) as a marker. The information on food products and diet compositions is shown in online Supplementary Tables S1 and S2. Ileal digesta samples were collected for 9 h on days 6 and 7, starting directly after the first meal of the day and were immediately frozen (–20°C). Pooled and mixed ileal samples from each pig were freeze-dried and ground (Retsch ZM200 centrifugal mill, Wageningen, The Netherlands) at 12 000 RPM over a 1-mm sieve before further use.

### Substrates

Ileal digesta samples from pigs (6–13, depending on the design of previous studies^(14,24)^) that ingested the same diet were pooled, based on an equal quantity of the indigestible marker (Ti). This resulted in thirty-one different ileal digesta samples from pigs consuming semipurified diets containing a wide range of protein sources including animal-based (AP: bovine collagen, cheddar, chicken, eggs, feta, fish and milk), grains (cornflakes, millet, oatmeal, rice crackers, rye bread, sorghum toasted wheat bread, wheat bran and wheat flour), legumes (black beans, chick peas, kidney beans, pigeon peas and roasted peanuts), fungi, algae and microorganisms (FAM: mushrooms, Quorn, seaweed, spirulina and yeast) and others (amaranth, buckwheat, linseed, potato and potato protein). Whey protein isolate (WPI, Fonterra, New Zealand) and WPI hydrolysate (WPIH, degree of hydrolysis = 25 %; Power Supplement, the Netherlands) were selected as controls. The N content of pooled ileal digesta samples, WPI and WPIH was analysed (online Supplementary Table S3) using the Dumas method (ISO 16634) at Wageningen University for calculating sample weight for the *in vitro* protein fermentation studies.

### Inoculum preparation

For preparation of the human and porcine inoculum, the method of Zhang *et al.* ^(9)^ was followed. Briefly, pooled fresh faeces collected from five self-reported healthy donors (25–30 years of age, no history of gastrointestinal disorders, recent antibiotic use (within three months prior to donation) or chronic illnesses) consuming their habitual diet or twenty growing pigs fed commercial diets were mixed under CO_2_ with 0·1 M phosphate buffered saline in a ratio of 1:5 (w/v) and 10 % (w/w) glycerol was added to the final mixture. The mixture was aliquoted in containers prefilled with CO_2_ and subsequently snap-frozen in liquid N. All containers were stored at −80°C until use. Before each incubation run, frozen inocula were thawed in a 39°C water bath and centrifuged at 200 × g for 10 min. The supernatant was collected and centrifuged again at 4816 × g for 10 min. The pellets containing the microbiota were collected and resuspended in the N-free buffer to a final faecal concentration of 2 %.

### *In vitro* protein fermentation

A precisely weighed amount of pooled ileal digesta, containing close to 10 mg N, was individually incubated (*n* 31) in one of three independent runs using human and porcine faecal inoculum. Within each run, blank bottles without added substrate as well as bottles containing 10 mg N from WPI and WPIH were incubated in triplicate. The *in vitro* protein fermentation procedure was performed as previously described^(9)^ with minor adjustments to allow a safe utilisation of human inoculum. Briefly, sealed bottles of 250 ml containing 60 ml of 2 % human or porcine faecal inoculum in a N-free buffer were prepared at the start of each run and incubated at 39°C until the addition of the test substrate. The buffer was supplemented with 21·56 g/l easily fermentable carbohydrates, including 8·6 g maltose (M5885), 4·32 g pectin from citrus peel (P9135), 4·32 g xylose (X1500, all from Sigma-Aldrich, Saint Louis, USA) and 4·32 g soluble potato starch (Paselli WA4, Avebe food, Veendam, The Netherlands). The timing of the addition of substrate to the buffer–faecal mixture was determined by monitoring the GP of the blank bottles at 39°C, which contained the same buffer–faecal mixture. This blank GP was recorded continuously using the method described by Cone *et al.* (1996; 2005)^(13,25)^ until it reached a plateau after 1–2 h. Subsequently, the ileal digesta and control substrates were added, bottles were closed and connected to the automatic gas recording equipment and incubated in water baths for 48 h with continuous recording of GP. The water level and temperature (39°C) in the water baths were maintained throughout the fermentation period.

### Curve fitting

Ileal digesta samples showed an S-shaped curve in cumulative GP, indicating a common pattern of this microbial process that fits with our assumption that after the addition of the sample to the pre-fermentation buffer–inoculum mixture, first the N from the substrate is accessible to the microbiota and after the substrate N is utilised, GP is driven by the accessibility of N through microbiota turnover.

The cumulative GP data for each bottle, from continuously recorded GP over 48 h, were used to calculate the: 1. lag time (T_lag_, h) of the start of fermentation (the time at which the cumulative GP of the substrate surpassed the cumulative GP of the blank within a run), 2. maximum GP rate (R_max_, ml/h) by dividing the gas released between two consecutive recorded time points by the time interval, 3. time when R_max_ occurred (T_Rmax_, h), 4. total GP generated from the protein source provided (GP_s_, ml/10 mg N) as determined by the model below, 5. time when GP_s_ occurred and microbiota turnover is assumed to start (T_GPs_, h) and 6. slope (ml/h) of the linear line fitted to the cumulative GP after T_GPs_. The model of Zhang *et al.* (2024)^(9)^ was used to fit the cumulative GP data of individual bottles:

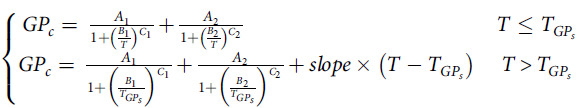




where *GP*_*c*_ **(**ml/10 mg N) denotes the amount of gas produced per 10 mg N of sample incubated (also corrected by the GP of the blank groups at T_lag_) at time *T* after T_lag_, A_i_ (ml/10 mg N) represents the asymptotic GP, B_i_ (h) is the time after incubation at which half of the asymptotic amount of gas has been formed and C_i_ is a constant determining the sharpness of the switching characteristic of the curve. The parameter *i* indicates the number of phases in the curve (*i* = 1, 2). The model was used to derive *GP*_*s*_, *T*_*GPs*_ and the slope.

### Statistical analyses

Differences of T_lag_, R_max_, T_Rmax_, GP_s_, T_GPs_ and slope between the thirty-one ileal digesta samples incubated with human and porcine inoculum were analysed using a mixed model in SAS 9.4 (SAS Inst. Inc.). In this model, dietary protein source and donor species of inocula were considered as fixed factors, the interaction (diet × donor-species) was examined and replication run was treated as a random factor. Similar analysis was done for WPI and WPIH. Further linear regression analysis was conducted for the GP parameters that showed a significant donor-species effect. Additionally, one-way ANOVA was used to compare the GP parameters between food categories for ileal digesta samples incubated with human inoculum. All residuals were normally distributed. Probability values < 0·05 were considered significant and values < 0·1 considered a trend. Group values were reported as means (standard error). Observations were excluded if bottle leakage was detected, as determined by comparisons with blank and replicate samples. Outliers for regression analysis were defined with Cook’s distance method using 0·129 (4/31) as a threshold.

## Results

### Gas production curves

**Figure 1.**
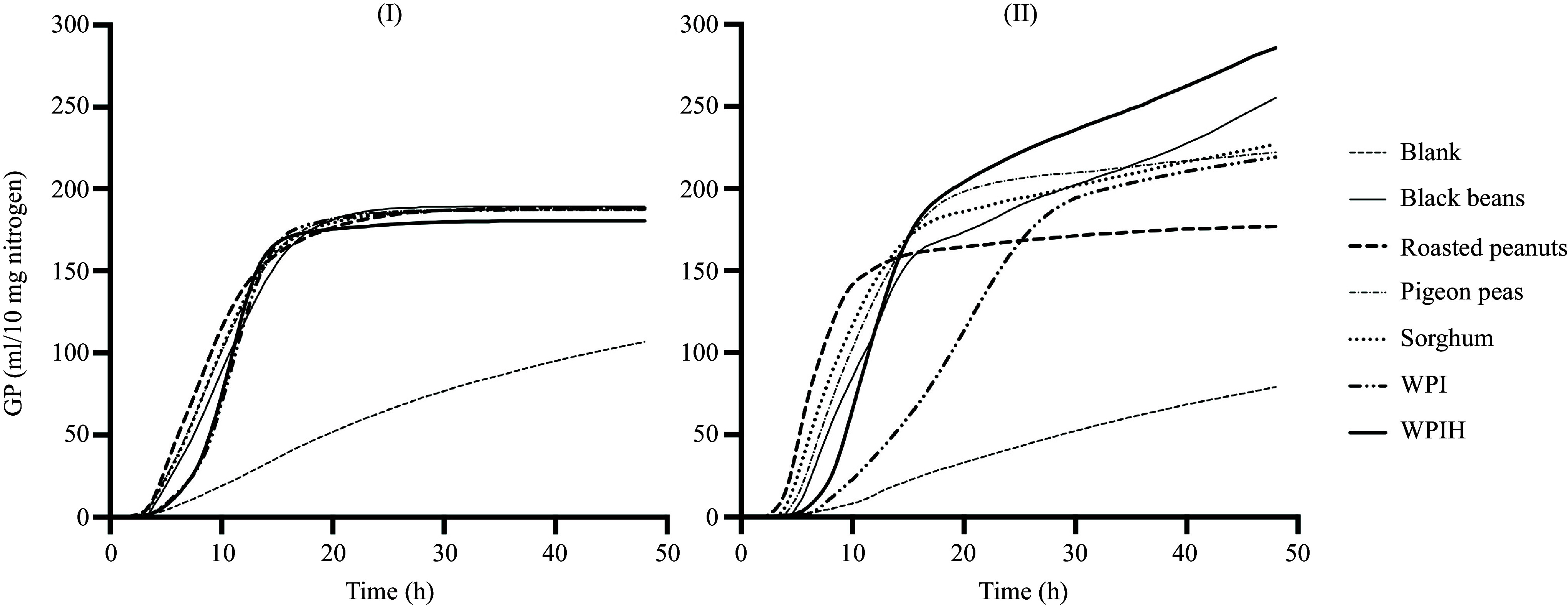
Measured 48 h *in vitro* cumulative gas production (GP) of ileal digesta from pigs fed four different human foods, whey protein isolate (WPI) and WPI hydrolysates (WPIH) using human (I) and porcine (II) faecal inoculum (blank). All samples contained 10 mg of nitrogen and mean values from three incubation runs were used.

**C**umulative GP curves of the diet-derived fermentable proteins from black beans, roasted peanuts, pigeon peas and sorghum, as well as WPI, WPIH and blank samples measured over 48 h using human and porcine faecal inoculum are shown as representative examples in Figure 1. Generally, the cumulative GP curves with human inoculum displayed less variation in their pattern compared to those incubated with porcine inoculum, especially after reaching GP_s_.

### Gas production parameters of foods with human faecal inoculum

**Figure 2.**
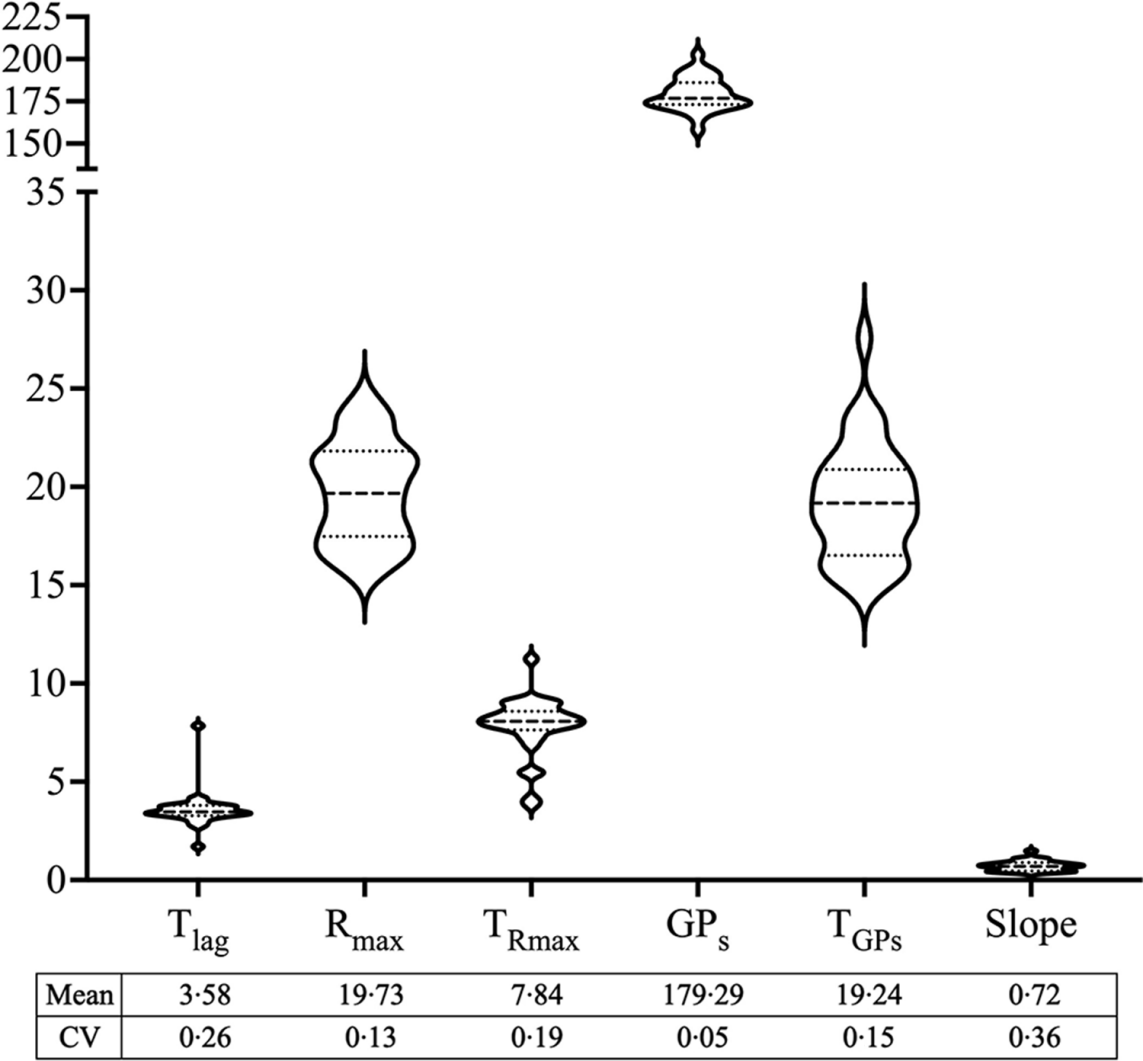
Overview of *in vitro* fermentation parameters of porcine ileal digesta (containing 10 mg nitrogen) originating from thirty-one different human foods incubated with human faecal inoculum. Data distribution, mean and coefficient of variation (CV) are shown for lag time (T_lag_, h), maximum gas production rate (R_max_, ml/h), time when maximum rate occurred (T_Rmax_, h), cumulative gas production of protein substrate determined by the model (GP_s_, ml/10 mg nitrogen), time when GP_s_ occurred (T_GPs_, h) and slope of the linear line of the model (slope, ml/h). Average value from three independent runs was used for each food. The shape of the violin plots illustrates the distribution of data points across the range of values. Symmetry or asymmetry in the plot shape indicates skewness or uniformity of the data distribution, while wider or narrower sections highlight regions of higher or lower data density, aiding in visual interpretation of variability.

**Figure 3.**
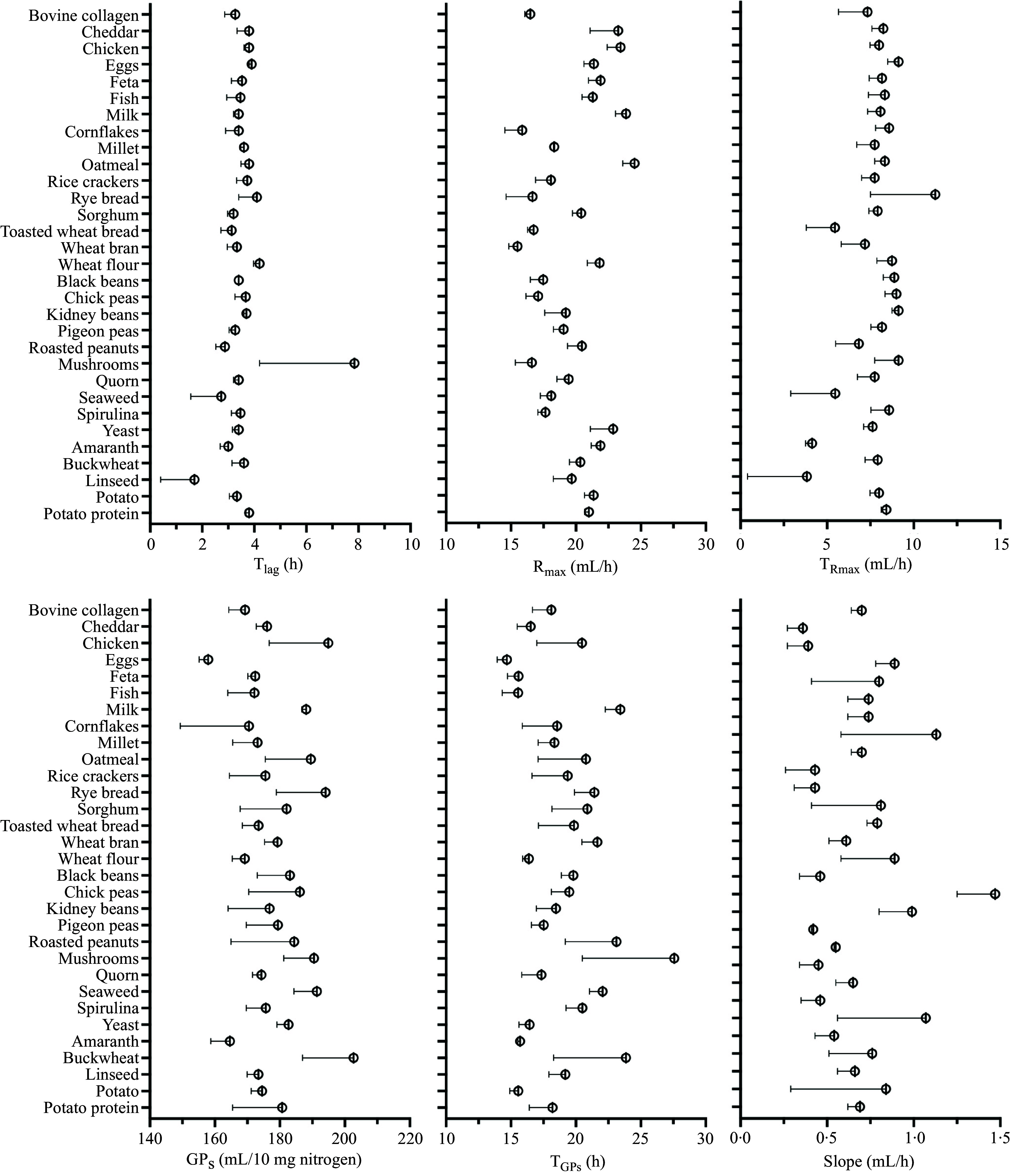
*In vitro* fermentation parameters of porcine ileal digesta samples containing 10 mg nitrogen, incubated with human faecal inoculum. Ileal digesta originated from previous studies investigating the digestibility of thirty-one different human foods. Values are means (se) of lag time (T_lag_), maximum gas production rate (R_max_), time when maximum rate occurred (T_Rmax_), cumulative gas production of protein substrate determined by the model (GP_s_), time when GP_s_ occurred (T_GPs_) and slope of the linear line of the model (Slope) of three incubation runs.

Violin plots visualising the distribution of GP parameters for the diet-derived fermentable proteins (*n* 31) incubated with human faecal inoculum are provided in Figure 2. Coefficient of variation of the parameters from the model fitted to the cumulative GP was relatively variable, with the slope having the highest and GP_s_ the lowest CV. Figure 3 presents the detailed information of the six parameters for the thirty-one individual foods. Significant effects of diet were found for all the parameters (*P* < 0·05), while the interaction between donor specie**s** and diet was significant for T_lag_, R_max_ and slope (*P* < 0·05).

The T_lag_ ranged from 1·7 (se 1·3) h for linseed- to 7·9 (se 3·7) h for mushroom-derived fermentable proteins with a mean of 3·6 (se 0·2) h. The values of R_max_ ranged from 15·5 (se 0·7) ml/h for wheat bran to 24·5 (se 0·9) ml/h for oatmeal, with the associated T_Rmax_ values ranging from 3·8 (se 3·4) h for linseed to 11·3 (se 3·8) h for oatmeal. Higher R_max_ values were found for oatmeal-, milk- and chicken-derived fermentable proteins (24·5 (se 0·9), 23·9 (se 0·8) and 23·4 (se 1·0) ml/h, respectively) compared to bovine collagen-, cornflakes- and wheat bran-derived (16·5 (se 0·4), 15·9 (se 1·3) and 15·5 (se 0·7) ml/h, respectively). The GP_s_ was found to be the lowest for egg- and highest for buckwheat-derived fermentable proteins (157·9 (se 2·7) and 202·7 (se 15·7) ml/10 mg N, respectively). Egg-derived fermentable proteins also had the lowest T_GPs_ (14·7 (se 0·7) h) while buckwheat- (23·9 (se 5·6) h) and mushroom-derived fermentable proteins (27·6 (se 7·1) h) had the second and first highest values. The slope ranged from 0·36 (se 0·09) to 1·5 (se 0·22) ml/h for cheddar- and kidney bean-derived fermentable proteins, respectively.

**Figure 4.**
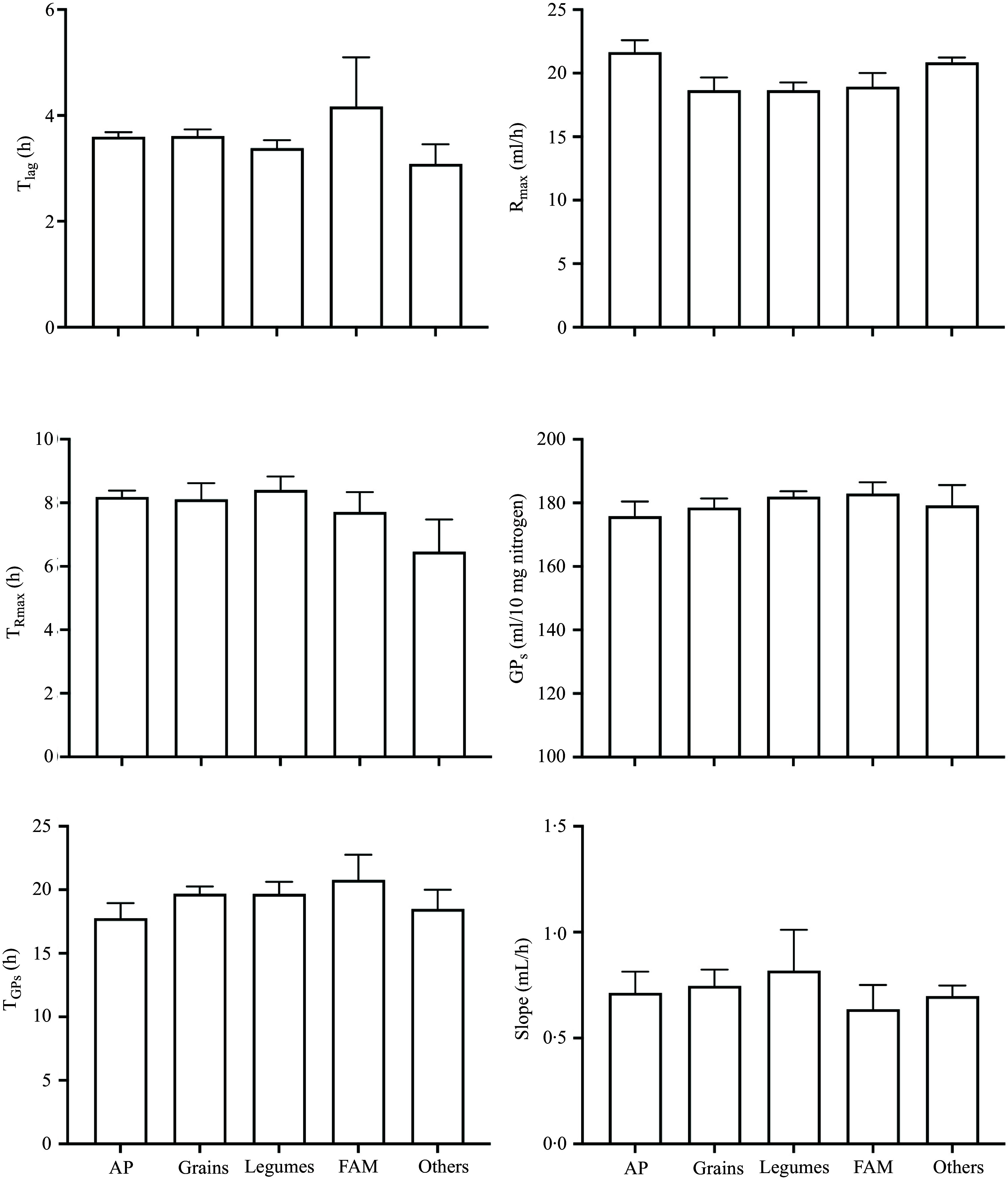
*In vitro* fermentation parameters of porcine ileal digesta samples (containing 10 mg nitrogen) incubated with human faecal inoculum in three independent runs. Ileal digesta originated from previous studies investigating the digestibility of thirty-one different human foods were grouped into animal proteins (AP), grains, legumes, fungi, algae and microorganisms (FAM) and others. Values are means (se) of lag time (T_lag_), maximum gas production rate (R_max_), time when maximum rate occurred (T_Rmax_), cumulative gas production of protein substrate determined by the model (GP_s_), time when GP_s_ occurred (T_GPs_) and slope of the linear line of the model (Slope) for each food category.

When the thirty-one foods were grouped into five categories, GP parameters showed no significant differences (Figure 4). Numerically, the highest values of R_max_ were observed for AP (21·7 (se 1·0) ml/h) and the lowest for grain (18·7 (se 1·0) ml/h) proteins.

### Donor-species effects on gas production parameters

The donor specie**s** of inoculum was found to significantly (*P* < 0·05) affect all fermentation parameters except for T_lag_. Nitrogen-containing components in the substrate in all groups started to be fermented around 3·6 h for both inocula. Lower R_max_ (19·4 (se 0·2) *v*. 21·8 (se 0·2) ml/h), higher T_Rmax_ (7·8 (se 0·2) *v*. 6·6 (se 0·2) h), lower GP_s_ (179 (se 1·6) *v*. 190 (se 1·7) ml/10 mg N), lower T_GPs_ (19·3 (se 0·4) *v*. 21·0 (se 0·4) h) and lower slope (0·7 (se 0·05) *v*. 1·6 (se 0·05) ml/h) values were found with human compared to porcine inoculum.

Comparisons of parameters for WPI and WPIH incubated with human and porcine faecal inoculum are shown in Table 1. Donor-species effect was significant for all parameters except for T_lag_. Higher R_max_ values were found for samples incubated with human inoculum, while a greater difference between WPI and WPIH was observed for porcine inoculum. WPI incubated with human inoculum showed a higher R_max_ and lower T_GPs_ value.

**Table 1.** *In vitro* fermentation parameters of whey protein isolate (WPI) and WPI hydrolysate (WPIH) containing 10 mg nitrogen incubated with human and porcine faecal inoculum

Parameter^*^	Human		Porcine		Pooled se	*P*-value^†^		
	WPI	WPIH	WPI	WPIH		Donor-Species	Substrate	Interaction
T_lag_	4·5	4·6	6·4	5·3	0·6	0·07	0·44	0·36
R_max_	27·4^b^	35·7^a^	12·5^c^	28·2^b^	1·0	< 0·0001	< 0·0001	0·006
T_Rmax_	11·2^b^	10·3^b^	21·0^a^	10·1^b^	0·9	0·0005	0·0001	0·0004
GP_s_	179^ab^	173^b^	196^ab^	209^a^	10·3	0·03	0·72	0·37
T_GPs_	17·6^b^	16·3^b^	29·7^a^	21·0^b^	1·3	0·0002	0·005	0·02
Slope	0·8^bc^	0·6^c^	1·4^b^	2·8^a^	0·2	< 0·0001	0·01	0·003

* Lag time (T_lag_, h), maximum gas production rate (R_max_, ml/h), time when maximum rate occurred (T_Rmax_, h), cumulative gas production of protein substrate determined by the model (GP_s_, ml/10 mg nitrogen), time when GP_s_ occurred (T_GPs_, h) and slope of the linear line of the model (slope, ml/h) during *in vitro* gas production.

† Mixed model (SAS 9.4) was used to examine the effects of donor-species, substrate and their interaction. Significant differences (*P* < 0·05) between values are indicated by different superscript letters.

### Predictivity of porcine inoculum

**Figure 5.**
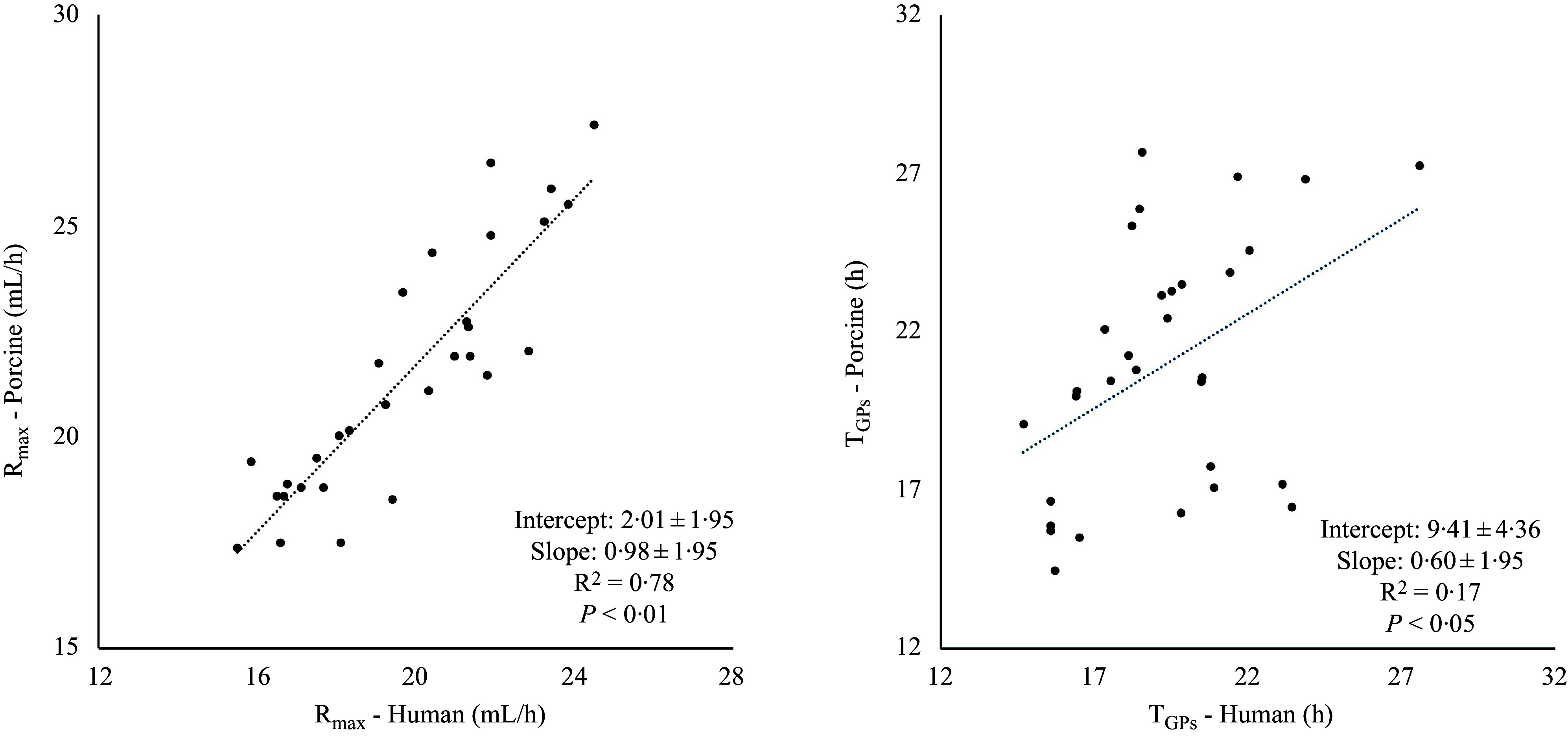
Scatter plot and linear regression coefficients (mean (sd)) of *in vitro* fermentation parameters of porcine ileal digesta incubated with human and porcine faecal inoculum. Parameters include the maximum gas production rate (R_max_) and the time at which the cumulative gas production of the protein substrate, as determined by the model, occurred (T_GPs_). Ileal digesta samples were obtained from growing pigs fed a wide range of human foods (*n* 30 for R_max_ and *n* 31 for T_GPs_). The average values from three incubation runs for each food were used.

GP parameters that showed a significant donor-specie**s** effect (all except T_lag_) were further examined through linear regression analysis. The R_max_ values of roasted peanuts were excluded from the regression analysis as the porcine inoculum had a Cook’s distance to the mean of the thirty-one foods larger than the threshold. A significant regression between donor-specie**s** was found for R_max_ and T_GPs_ (Figure 5) but not for other variables (T_Rmax_, GP_s_ and slope). For R_max_, the slope of the linear line was not significantly different from 1·0, while this was not the case for T_GPs_.

## Discussion

### Protein fermentation kinetics of the human foods

Despite growing interest in the gut health implications of dietary proteins, the fermentation potential of undigestible proteins in different foods remains largely unexplored. In this study, we utilised an *in vitro* system to investigate the GP kinetics of diet-derived fermentable proteins from a diverse range of human foods (*n* 31). By incubating ileal digesta samples, considering both undigested dietary proteins and endogenous proteins (basal and specific) as a combined proteinaceous source, a significant food effect was found, indicating that foods differ in their protein fermentation potential.

Fermentation kinetics are greatly affected by both the microbial composition and the type of substrate available. Studies examining human and porcine fermentation at the ileal level have highlighted the critical roles of these factors, often demonstrating that the substrate source has a more substantial impact^(26,27)^. Our results demonstrated significantly different GP kinetics across the various foods tested. Among the GP parameters, R_max_ was particularly noteworthy as a sensitive indicator of N accessibility in the diet-derived fermentable proteins. This finding aligns with our previous studies^(8,9)^ and the current data on WPI and WPIH, which indicates that R_max_ is closely related to protein composition, structure and the level of hydrolysis. In all the samples, R_max_ showed higher values for oatmeal-, milk- and chicken-derived fermentable proteins compared with bovine collagen-, cornflakes- and wheat bran-derived. Meanwhile, GP_s_ exhibited high consistency (179·3 (se 1·7) ml/10 mg N, CV = 5·4 %) indicating that microbiota tended to maximally utilise the provided 10 mg N across different foods. Conversely, the slope displayed high relative variability (CV = 36 %), which may indicate differences in microbial composition and turnover rates resulting from different proteins. This variability could also reflect potential differences in microbial enzymes, metabolic pathways and the influence of dietary components, such as antimicrobial compounds. Overall, these observations highlight the diverse metabolic activities of human microbiota when exposed to different diet-derived fermentable proteins.

The varied N accessibility can alter the gut microbial diversity and composition and, thereby, the fermentation characteristics^(28,29)^ and metabolite profiles in the gut and faeces^(30–32)^. This may be related to the different AA profiles in the samples, which are preferred by different bacterial groups and utilised differently^(5,33)^. A previous study comparing *in vitro* digested rice, oat, chicken, pork and beef found significant differences in microbial compositions and branched-chain fatty acid levels after 48-hour fermentation^(28)^. However, the latter study used initial weight rather than our N-standardised method and did not account for endogenous proteins. When equal amounts of N are used, the endogenous proteins in ileal digesta—known to be highly fermentable^(9)^—will contribute to different degrees, depending on dietary N digestibility. For instance, foods that are highly digestible result in a limited amount of dietary N but a higher ratio of endogenous N in the ileal digesta. Therefore, comparing protein source for their *in vivo* protein fermentation potential should be conducted on an equal N basis. This way, by taking into account apparent and true ileal digestibility using the DIAAS methodology, a more accurate *in vitro* dietary protein fermentation potential can be calculated as was previously done for porcine dietary protein sources^(8)^.

Interestingly, considerable sex-dependent influences on the microbial shift and metabolite profile during *in vitro* fermentation of different dietary protein sources were observed in humans in a recent study^(34)^. For instance, male-associated microbiota produced more p-cresol from non-animal-derived proteins compared to female-associated microbiota, while microbiota originating from females produced more ammonia from animal-derived proteins compared to males. Thereby, population-dependent dietary suggestions were recommended by the authors^(34)^. To enhance the accuracy of *in vitro* protein fermentation potential assessments across different populations, sex-specific inoculum should be considered in experimental designs.

Overall, to the best of our knowledge, this is the first study to report the fermentation potential of diet-derived fermentable proteins of a variety of a large number of human foods. These findings may contribute to providing appropriate dietary advice and guidelines to reduce the risks associated with detrimental impacts of protein fermentation in humans.

### Comparison between food categories

In the current study, no differences were observed for any of the GP parameters when the different foods were grouped into the categories (AP, grains, legumes, FAM and others). Only a tendency (*P* = 0·07) for a higher R_max_ in ileal digesta originating from AP compared to legume and grain proteins was observed per 10 mg of N. This lack of significance is potentially due to the high variation of individual foods within each category. For instance, within the grains, oatmeal-derived fermentable proteins showed the highest R_max_ while wheat bran-derived fermentable proteins exhibited the lowest among the thirty-one foods. In the category of AP, undigested and endogenous proteins originating from the ingestion of milk and chicken showed a much higher R_max_ than bovine collagen. Similarly, while significant differences were found for branched-chain fatty acid within cereal protein groups and AP groups^(28)^, the two groups had comparable average levels due to large variation between rice and oat. Also, the greater complexity and functional diversity of the faecal microbiota^(35)^ suggest enhanced fermentation capabilities. The microbiota can utilise a wider range of substrates, leading to more varied and efficient fermentation processes and may exhibit a less pronounced substrate effect when comparing broad food categories.

Another potential reason for the lack of differences between categories is that the current categorisation does not effectively distinguish the fermentation characteristics of different samples, especially for the ‘other’ category. For instance, potatoes are primarily composed of carbohydrates, particularly starch, while potato protein is isolated from potatoes and is rich in AA. Therefore, it is difficult to extrapolate the results in our study to other findings where only one repetition was used for each food category^(36)^ and multiple sources per food category is recommended to be included in future studies on protein fermentation. Overall, comparisons between individual foods, rather than broad food categories, may offer more valuable insights into protein fermentation dynamics.

Despite comparable *in vitro* fermentation potential, differences in protein digestibility between food categories can significantly impact overall protein fermentation in the gut under *in vivo* conditions. For example, AP demonstrated the highest true ileal digestibility among all categories (data not shown), resulting in less undigested protein reaching the large intestine for fermentation. All other study procedures, such as cannula location, were standardised throughout the experiment. However, a comparable N concentration was found in the ileal digesta samples across categories. This similarity may be due to varied contributions from endogenous proteins, which offset differences in dietary protein digestibility. Therefore, examining N flow in the gut may be the next step in applying *in vitro* findings to the *in vivo* fermentation potential of foods.

### Human and porcine microbiota as inoculum

When the same ileal digesta samples were incubated with porcine faecal inoculum, larger variation in GP patterns between samples was detected, compared to human inoculum. In general, human inoculum resulted in slower and less GP compared to porcine inoculum, indicating a lower fermentative activity in the human microbiota. The nonsignificant difference in T_lag_ observed between inocula indicates a comparable start of fermentation, while the lower R_max_, GP_s_, T_GPs_ and higher T_Rmax_ values with human inoculum indicate that human faecal microbiota can utilise protein substrates less extensively and efficiently than porcine faecal microbiota. Lower slope values observed for human microbiota at the end of fermentation also suggested a less active community. The similarity in GP parameters of porcine faecal inoculum compared to human inoculum was evaluated through linear regression analysis. A significant linear regression was only found for R_max_ and T_GPs_, indicating limitations in the overall applicability using porcine inoculum as a substitute for humans.

Similar, significant donor-species effects were observed for both WPI and WPIH across all parameters, except for T_lag_. Conversely, higher R_max_ values were recorded with human inoculum, suggesting that human faecal microbiota can utilise the highly soluble and digestible protein sources more quickly than porcine microbiota. Despite this, the total level of protein degradation, as indicated by the GP_s_, was comparable between the two. This indicates that while the rate of protein fermentation may differ, the extent of fermentation achievable by porcine inoculum can mirror that of human inoculum under certain conditions.

Apart from donor-specie**s** differences, we also observed interaction effects for T_lag_, R_max_ and slope between diet and donor-specie**s** with both ileal digesta and pure protein sources. These interactions suggest that certain protein sources may be fermented differently depending on whether the microbiota are of human or porcine origin. Similar interactions were also observed in fibre fermentation, as different GP parameters were found for various dietary fibres between inocula from humans and pigs^(37)^.

Previous studies have shown that the concentration of specific bacterial populations varies between humans and pigs. For example, *Bifidobacterium* species are more abundant in humans compared to pigs^(38)^. Another study found that human inoculum has a greater ability to ferment resistant starch, while porcine inoculum is more effective at fermenting cellulose^(37)^. In the study using arabinoxylan and galactoxyloglucan by Feng *et al.* (2020)^(39)^, differences in growth promotion of bacterial groups between humans and pigs were found during *in vitro* fermentation. These findings highlight the importance of understanding the specific microbial profiles involved in fermentation processes. The promotion of specific bacterial groups is likely also the explanation for the difference observed for the substrate utilisation in this study. This aligns with findings from previous research demonstrating that gut microbiota composition can significantly impact nutrient degradation patterns^(40)^. Furthermore, the significant lower turnover rate observed with the human inoculum at the end of incubation suggests human inoculum might contain microbial communities that are only highly efficient at quickly utilising substrate N, leading to rapid depletion and lower subsequent activity. In contrast, porcine inoculum might have a broader range of microbes capable of sustaining activity through endogenous substrate utilisation and biomass recycling, resulting in continuous turnover after substrate N is utilised. Microbial analyses, particularly for N-utilising species such as *Clostridium* and *Streptococcus*,^(5)^ were not performed in the current study. Including such analyses for microbial profiles of inocula before and after fermentation in future studies could provide further insights into the observed differences. Another explanation is that the endogenous proteins of humans and pigs are different, and the microbiota capable of degrading these endogenous proteins exhibit species-specificity^(41)^. Additionally, it is possible that porcine microbiota is more capable of digesting insoluble proteins in the samples. Porcine microbiota may have evolved to degrade such proteins more efficiently compared to humans, who typically consume more refined and processed proteins.

### Limitations and future directions

In the current study, a high level of easily fermentable carbohydrates and limiting N conditions were employed to ensure the direct reflection of N availability to microbiota. However, the specific structure and accessibility of proteins within each unique food matrix may still influence their availability for microbial fermentation. Moreover, this differs from *in vivo* conditions. In this study, the semi-synthetic experimental diets with only one protein source are different from the average human diets that are in Western countries mixtures of protein sources. It is yet unknown how mixtures of protein sources influence protein fermentation. When consuming the same food groups, the fibre content may play a significant role *in vivo*, as it is well-known for its effects on suppressing protein fermentation^(42)^. Furthermore, given the acknowledged variation in microbiota and microbial activity between different intestinal segments and faeces^(43)^, using faecal inoculum may not be representative for other segments of the intestinal tract^(44,45)^, and protein fermentation can already happen in the upper gastrointestinal tract of pigs^(46)^. Faecal microbiota may slightly overestimate *in vivo* fermentation, as observed in dogs^(45)^. Therefore, the results obtained with faecal inoculum in the current study require validation with *in vivo* studies for further application like dietary guidance.

Although growing pigs have been proven to be a good model for protein/AA digestibility in adult humans^(14,47)^, ileal digesta obtained from pigs may not fully resemble human digesta when used for *in vitro* protein fermentation due to differences in endogenous losses. With a protein-free diet, studies have shown that the level of endogenous protein in the ileal digesta of adult humans is significantly higher compared to that in growing pigs^(14,16)^. Although different durations of protein-free feeding were applied (7 d for pigs, while only a single meal was provided on two mornings for humans), correction for these differences in endogenous losses does make the ileal digestibility of dietary protein and AA between growing pigs and humans comparable^(14)^.

In the current study design, ileal digesta were incubated with both inocula at the same temperature (39°C) to ensure a completely randomised design and allow direct comparison of parameters. The same temperature was used in a previous study comparing twelve different dietary fibre with human and porcine inoculum^(37)^. This temperature is typical for the porcine large intestine but is slightly higher than the typical human body temperature (37°C), which might influence microbial activity and composition. Studies have shown that the growth of some bacterial groups, such as *E. coli* and *Bacillus*, was not affected when the temperature was increased from 37 to 39°C^(48)^, while the abundance of *Firmicutes* was enhanced at a higher temperature of 44°C^(49)^. However, these studies used samples obtained from porcine faeces and chicken neck skin rather than specific strains from humans. Additionally, human gut pathogens such as *Clostridium difficile* have been found to grow equally well at 37 and 41°C *in vitro* ^(50)^. Although a slight difference in temperature has not specifically been examined for *in vitro* GP studies, the best temperature would be close to the natural body temperature. In this study, the low turnover rate at the end of the cumulative GP curve for human faecal microbiota suggests an impaired microbiota community compared to porcine faecal microbiota. However, it is unclear if this is due to the elevated temperature or higher sensitivity to accumulated harmful metabolites during fermentation or simply the different microbiota composition. Overall, using the appropriate temperature for each species separately is recommended in future research to accurately examine the uncoupled responses.

Lastly, standardised frozen inocula were used (after thawing) in this study for higher comparability and replicability. The impact of pooling, freezing and long-term storage on inoculum activity has often been a point of discussion^(51–54)^. Although studies have found that human faeces preserved at –80°C provided a consistent and stable inoculum for performing batch fermentations for up to 3 years, particularly regarding metabolite production^55)^, the abundance and diversity of microbiota can still change during storage and impact the GP profile^(52,54)^. For instance, the storage of equine faeces for 24, 48, 72 h or 7 d at –20°C showed varied rates of GP during *in vitro* experiments^(53)^. These findings underscore the importance of carefully selecting and standardising storage conditions to ensure accurate and reliable results in studies investigating microbiota and its fermentation capabilities.

### Conclusion

This is the first report on the *in vitro* fermentation potential of undigested protein in thirty-one foods consumed by humans using ileal digesta and compared the use of human and porcine inocula. With human inoculum, marked differences were observed in the fermentation kinetic parameters of the ileal digesta samples from the wide coverage of food items studied. The data provided in conjunction with DIAAS estimates allows for the calculation of a protein fermentation potential value of foods. The R_max_ between human and porcine faecal inoculum are directly comparable but other fermentation parameters are not. Human faecal inoculum remains crucial for accurately predicting *in vitro* protein fermentation potential and outcomes relevant to humans.

## Supporting information

Zhang et al. supplementary material Zhang et al. supplementary material

## Supplementary material

For supplementary material/s referred to in this article, please visit https://doi.org/10.1017/S0007114525103541
