# Supplementary material for: In vitro fermentation potential of diet-derived fermentable proteins of thirty-one human foods
Source: Br J Nutr. 2025 Jun 3;133(11):1456–65. doi: 10.1017/S0007114525103541 (PMC12303725; doi:10.1017/S0007114525103541)
Supplement: Zhang et al. supplementary material [file S0007114525103541sup001.docx]

***In Vitro* Fermentation Potential of Diet-Derived** **Fermentable Proteins of 31 Human Foods**

Hanlu Zhang^a,b^, John W. Cone^a^, Arie K. Kies^c^, Wouter H. Hendriks^a^ and Nikkie van der Wielen^a,d,*^

^a^ Animal Nutrition Group, Department of Animal Sciences, Wageningen University & Research, Wageningen, The Netherlands;

^b^ State Key Laboratory of Animal Nutrition, College of Animal Science and Technology, China Agricultural University, Beijing, China;

^c^ ArieKiesAdvies, Druten, The Netherlands;

^d^ Division of Human Nutrition and Health, Department of Agrotechnology and Food Sciences, Wageningen University & Research, Wageningen, The Netherlands.

**^*^**Correspondence: [nikkie.vanderwielen@wur.nl](mailto:nikkie.vanderwielen@wur.nl)

**Running title:** Fermentation Potential of Undigested Protein of Human Foods

**Keywords:** Undigested protein, *in vitro* fermentation, human inoculum, porcine model, gas production

**Supplementary Materials**

The following description is adapted from previous studies where the ileal digesta samples used in the current experiment were collected.

***Preparation of test foods***

The origin of all foods is specified in Table S1.

Spirulina, yeast, linseed (broken), wheat flour, potato protein and bovine collagen were stored in serving bins at room temperature, milk and cheddar (shredded) were stored separately in the fridge until serving, they required neither cooking nor processing before feeding.

Sorghum flour was mixed with water (1:4). The resulting mixture was boiled on a stovetop for approximately 20 min until a thick, paste-like consistency was achieved.

Kidney beans, rice crackers, cornflakes and feta cheese were cut into ~0.2 cm pieces using a cutter (Mado Supra 50). Seaweed was cut into ~0.5 cm and subsequently ground to powder. After processing, seaweed, rice crackers, and cornflakes were stored in serving bins at room temperature, kidney beans were stored in serving bins in the fridge and feta was stored in closed bags in a freezer (-20°C) if not consumed within 2-3 days.

For the toasted wheat bread, the bread dough was prepared and baked by the Department of Food Science and Human Nutrition Pilot Processing Plant at the University of Illinois at Urbana-Champaign and followed a standardized recipe. Sucrose was dissolved in the warmed water and active dry yeast was added to this solution. After yeast activity was confirmed, salt, butter, and flour were added to the solution. Titanium dioxide was mixed into the solution for the bread to be consumed by pigs as indigestible markers. Dough was then kneaded in a commercial floor mixer (Hobart Legacy® Mixer, Troy, OH) and portioned into 23 × 13 × 6 cm baking pans where it was allowed to proof for 1 h before being baked at 175°C for 35 min. After baking, loaves were cooled to room temperature and then frozen. Immediately before feeding, loaves were thawed, sliced, and toasted lightly.

Frisian-style rye bread was baked at Wageningen University by filling bread tins with a mixture of rye kernels and tap water in a 30:70 ratio. These tins were tightly sealed and placed in a preheated Rototherm Green convection oven at 170°C with 2.5 L of steam applied, and baked for 16 hours at 120°C. Another batch of rye bread dough was prepared by mixing soaked and untreated rye kernels in a 1:3 ratio with water, salt, TiO_2_, and PEG8000. This dough was mixed for 15 minutes, then transferred to tightly sealed bread tins and baked under the same conditions as the first batch. After baking, the rye breads were cooled, frozen at -18°C until testing, and then cut into 1 cm pieces.

Black beans and pigeon peas were procured in the dry form and prepared for feeding according to IDRC (1977)^(1)^. They were soaked in water at room temperature for 18 h prior to cooking. After soaking, they were strained and weighed. Tablet salt was added, at 720 mg per 100 g beans, in solution with water, and beans were then cooked in a pressure cooker at a pressure of approximately 100 kPa and a temperature of 121°C (20 min for black beans and 10 min for pigeon peas).

Peanuts (blanched peanuts, deshelled, dehulled, roasted) were coarsely ground by Department of Food Science and Human Nutrition Pilot Processing Plant at the University of Illinois at Urbana-Champaign before being shipped to each laboratory. Peanuts were broken with a hammer mill into 2-5 mm pieces before consumption.

Potatoes were pressure cooked (2 L water per 5 kg potatoes) for 10 min then cut into 0.2 cm pieces. They were stored in closed buckets in a freezer (-20°C) until use if not consumed within 2-3 days.

Kellogg’s® All-Bran® was blended with water to achieve a smooth consistency.

Mushrooms were cut in slices and steamed for 6 min in an oven, and then cut into ~0.2 cm pieces. Buckwheat, amaranth and millet, and oatmeal were simmered with water, at ratios or 7:20, 1:2, and 4:25 g/mL, respectively, for 25 min (15 for oatmeal) in an oven. After cooling down, they were stored in closed bags in the freezer if not consumed within 2-3 days.

Quorn, chicken, and fish (tilapia) were mixed with water at a ratio of 3:5 (g/mL) and cooked in an oven. After boiling, the oven heat was reduced to low for Quorn and fish to simmer for 9-10 min and was reduced to medium for chicken to simmer for 20-25 min until it was cooked through. Eggs were placed in an oven tray with holes and cooked by setting the program in oven to ‘Steam eggs’. After cooling down and peeling the eggs, they were cut into ~0.2 cm pieces and stored in closed bags in the freezer if not consumed within 2-3 days.

Test diets were formulated in which the test food was the only source of protein, and the final diets contained 100 g crude protein/kg (Table S2). The test diets also contained purified maize starch, sucrose, vitamins and minerals, refined vegetable oil (human food quality), purified cellulose and TiO_2_. A protein-free diet was also formulated.

***Feeding protocols***

Procedures followed the protocol of Hodgkinson et al. (2020)^(2)^. Healthy female pigs of a commercial maternal line (Landrace/Large White) were used. Bodyweight at surgery was at least 30 kg and maximum bodyweight at the end of the study was 100 kg. Throughout the study, the daily dietary ration for each pig was 0.08 × metabolic bodyweight (kg^0.75^) calculated on a DM basis. The daily ration was given in 2 equal meals 9 hours apart (8:00am and 5:00pm). Test cycles for each test food had a 7-day duration. At dietary change from basal diet to test diets, pigs were weighed, and their daily ration adjusted according to their bodyweight. For each experimental diet, the number of pigs used ranged from 6 to 13, depending on the design of each study. Pigs were allotted to their test cycles according to a Latin Square or an incomplete Latin Square (Youden Square) with diets and periods comprising the rows and the columns of the squares, respectively. The initial 5 days of each test were the adaptation period to the diet.

Digesta were collected via the cannula for 9 hours on days 6 and 7 starting immediately after the first meal of the day. Small plastic bags were attached to the cannula barrel using a zip tie or elastic band. Bags were replaced whenever filled with digesta or at least once every 30 min and the digesta were immediately frozen (-20°C). Digesta were thawed but maintained at < 4°C. After pooling and mixing the digesta, a subsample of the digesta from each pig and diet was collected and freeze-dried.

| Table S1. Product information of the test foods. | | |
| --- | --- | --- |
| Food category | Food | Brand |
| Animal-based | Bovine collagen | Dat-Schaub, Poland |
|  | Cheddar | Albert Heijn, The Netherlands |
|  | Chicken | Chicken breast, Local butcher |
|  | Eggs | Free range eggs medium, Albert Heijn, The Netherlands |
|  | Feta | Dodoni Feta cheese, The Netherlands |
|  | Fish | Tilapia filet, Albert Heijn, The Netherlands |
|  | Milk | Campina semi-skimmed milk, pasteurised, The Netherlands |
| Grains | Cornflakes | Kellogg's Cornflakes, Kellogg’s, USA |
|  | Millet | Millet, Smaakt bio, The Netherlands |
|  | Oatmeal | Quaker Oatmeal, Pepsico, USA |
|  | Rice crackers | Ricetoast natural, Van der Meulen, The Netherlands |
|  | Rye bread | Baked at Wageningen University (See Supplementary Material) |
|  | Sorghum | Sorghum flour, United States of America |
|  | Toasted wheat bread | Baked at University of Illinois (See Supplementary Material) |
|  | Wheat bran | Kellogg’s All Bran Pty. Ltd, Australia |
|  | Wheat flour | Patent wheat flour, Albert Heijn, The Netherlands |
| Legumes | Black beans | Harvest North, Henshall, Canada |
|  | Chickpeas | Sofia, Italy |
|  | Kidney beans | Kidneybonen, Albert Heijn, The Netherlands |
|  | Pigeon peas | Davis Food Ingredients, New Zealand |
|  | Roasted peanuts | Argentina |
| Fungi, algae, and microorganisms | Mushrooms | White Mushrooms (*Agaricus bisporus*), Local greengrocer |
|  | Quorn | Quorn Pieces, Quorn Foods, United Kingdom |
|  | Seaweed | Yakisushinori A grade, Yama, Japan |
|  | Spirulina | Bio Spirulina, Eco Mundo, The Netherlands |
|  | Yeast (inactive) | Speerstra, The Netherlands |
| Others | Amaranth | Biologic amaranth, Ekoplaza, The Netherlands |
|  | Buckwheat | Buckwheat, Smaakt bio, The Netherlands |
|  | Linseed | Biologic Linseed Broken, Albert Heijn, The Netherlands |
|  | Potato | Agria, The Netherlands |
|  | Potato protein | Coagulated potato protein, Avebe, The Netherlands |

| Table S2. Ingredient composition of the experimental diets (g/kg DM) used in the two different pig experiments with the same protocol for ileal digesta collection^1^. | | | | | | | | | | | | | | | | | |  |
| --- | --- | --- | --- | --- | --- | --- | --- | --- | --- | --- | --- | --- | --- | --- | --- | --- | --- | --- |
| Diet | Test protein | Purified maize starch | Purified cellulose | Rapeseed oil | Sucrose | Premix (vit+min)^2^ | Dicalcium phosphate | Magnesium oxide | CaCO_3_ | K_2_CO_3_ | NaHCO_3_ | Salt (NaCl) | Salt extra | Celite | Titanium dioxide | PEG8000^3^ | Sum | |
| Bovine collagen | 102 | 662 | 30 | 50 | 100 | 1.5 | 25 | 1 | 3 | 7 | 3 | 0 | 4 | 7.5 | 4 | 0 | 1000 | |
| Cheddar | 179.3 | 588 | 30 | 50 | 100 | 5 | 25 | 1 | 3 | 7 | 3 | 0 | 0 | 0 | 4 | 5 | 1000 | |
| Chicken | 107 | 656 | 30 | 50 | 100 | 5 | 25 | 1 | 3 | 7 | 3 | 0 | 4 | 0 | 4 | 5 | 1000 | |
| Eggs | 180.6 | 582 | 30 | 50 | 100 | 5 | 25 | 1 | 3 | 7 | 3 | 0 | 4 | 0 | 4 | 5 | 1000 | |
| Feta | 267 | 503 | 30 | 50 | 100 | 5 | 25 | 1 | 3 | 7 | 0 | 0 | 0 | 0 | 4 | 5 | 1000 | |
| Fish | 113 | 650 | 30 | 50 | 100 | 5 | 25 | 1 | 3 | 7 | 3 | 0 | 4 | 0 | 4 | 5 | 1000 | |
| Milk | 323.6 | 443 | 30 | 50 | 100 | 5 | 25 | 1 | 3 | 7 | 3 | 0 | 0 | 0 | 4 | 5 | 1000 | |
| Cornflakes | 947 | 0 | 0 | 0 | 0 | 5 | 25 | 1 | 3 | 7 | 3 | 0 | 0 | 0 | 4 | 5 | 1000 | |
| Millet | 881.8 | 0 | 30.6 | 30.6 | 0 | 5 | 25 | 1 | 3 | 7 | 3 | 0 | 4 | 0 | 4 | 5 | 1000 | |
| Oatmeal | 760.5 | 0 | 91.6 | 91.6 | 0 | 5 | 25 | 0 | 3 | 7 | 3 | 0 | 4 | 0 | 4 | 5 | 1000 | |
| Rice Crackers | 947 | 0 | 0 | 0 | 0 | 5 | 25 | 1 | 3 | 7 | 3 | 0 | 0 | 0 | 4 | 5 | 1000 | |
| Rye bread | 927.9 | 0 | 9.5 | 9.5 | 0 | 5 | 25 | 1 | 3 | 7 | 3 | 0 | 0 | 0 | 4 | 5 | 1000 | |
| Sorghum | 945 | 0 | 0 | 0 | 0 | 1.5 | 25 | 0 | 3 | 7 | 3 | 0 | 4 | 7.5 | 4 | 0 | 1000 | |
| Toasted wheat bread | 949 | 0 | 0 | 0 | 0 | 1.5 | 25 | 0 | 3 | 7 | 3 | 0 | 0 | 7.5 | 4 | 0 | 1000 | |
| Wheat bran | 803 | 0 | 72.5 | 72.5 | 0 | 1.5 | 25 | 0 | 3 | 7 | 3 | 0 | 1 | 7.5 | 4 | 0 | 1000 | |
| Wheat flour | 812 | 0 | 65 | 65 | 0 | 5 | 25 | 1 | 3 | 7 | 3 | 0 | 4 | 0 | 4 | 5 | 1000 | |
| Black beans | 488 | 277 | 30 | 50 | 100 | 1.5 | 25 | 0 | 3 | 7 | 3 | 4 | 0 | 7.5 | 4 | 0 | 1000 | |
| Chickpeas | 522 | 247 | 30 | 50 | 100 | 1.5 | 25 | 0 | 3 | 7 | 3 | 0 | 0 | 7.5 | 4 | 0 | 1000 | |
| Kidney beans | 380.8 | 392 | 30 | 50 | 100 | 5 | 25 | 1 | 3 | 0 | 0 | 0 | 4 | 0 | 4 | 5 | 1000 | |
| Pigeon peas | 488 | 277 | 30 | 50 | 100 | 1.5 | 25 | 0 | 3 | 7 | 3 | 4 | 0 | 7.5 | 4 | 0 | 1000 | |
| Roasted peanuts | 357 | 408 | 39 | 50 | 100 | 1.5 | 25 | 0 | 3 | 7 | 3 | 0 | 4 | 7.5 | 4 | 0 | 1000 | |
| Mushrooms | 253.5 | 517 | 30 | 50 | 100 | 5 | 25 | 1 | 3 | 0 | 3 | 0 | 4 | 0 | 4 | 5 | 1000 | |
| Quorn | 181 | 582 | 30 | 50 | 100 | 5 | 25 | 1 | 3 | 7 | 3 | 0 | 4 | 0 | 4 | 5 | 1000 | |
| Seaweed | 191 | 587 | 30 | 50 | 100 | 5 | 25 | 0 | 3 | 0 | 0 | 0 | 0 | 0 | 4 | 5 | 1000 | |
| Spirulina | 140.5 | 623 | 30 | 50 | 100 | 5 | 25 | 1 | 3 | 7 | 3 | 0 | 4 | 0 | 4 | 5 | 1000 | |
| Yeast | 207 | 569.5 | 30 | 50 | 100 | 5 | 12.5 | 1 | 12 | 0 | 0 | 0 | 4 | 0 | 4 | 5 | 1000 | |
| Amaranth | 592.4 | 172 | 30 | 50 | 100 | 5 | 25 | 0 | 3 | 7 | 3 | 0 | 4 | 0 | 4 | 5 | 1000 | |
| Buckwheat | 675.7 | 0 | 133.7 | 133.7 | 0 | 5 | 25 | 1 | 3 | 7 | 3 | 0 | 4 | 0 | 4 | 5 | 1000 | |
| Linseed | 458.7 | 305 | 30 | 50 | 100 | 5 | 25 | 0 | 3 | 7 | 3 | 0 | 4 | 0 | 4 | 5 | 1000 | |
| Potato | 924.2 | 0 | 9.4 | 9.4 | 0 | 5 | 25 | 1 | 3 | 7 | 3 | 0 | 4 | 0 | 4 | 5 | 1000 | |
| Potato protein | 115 | 648 | 30 | 50 | 100 | 5 | 25 | 1 | 3 | 7 | 3 | 0 | 4 | 0 | 4 | 5 | 1000 | |
| ^1^For diets with low protein content ingredients (rice crackers, dried dates, cornflakes and corn tortillas), an animo acids mixture were added on day 1to 4.  ^2^Vitamin and mineral premix in final diet (per kg dry matter): Cu 20 mg; I 1.3 mg; Fe 123 mg; Mn 60 mg; Se 0.3 mg; Zn 125 mg; niacin 44 mg; cobalamin 0.03 mg; pantothenic acid 23 mg; riboflavin 6.5 mg; phytonadione 1.4 mg; biotin 0.44 mg; retinol 10622 IU; cholecalciferol 1660 IU; d,l-α-tocopherol 66 IU; pyridoxine 0.98 mg; folate 1.6 mg; thiamine 1.1 mg.  ^3^PEG8000: Polyethylene glycol 8000 | | | | | | | | | | | | | | | | | | |
|  | | | | | | | | | | | | | | | | | |  |

| Table S3. Nitrogen content of substrates used in the *in vitro* gas production tests. Ileal digesta samples were pooled from 6 to 13 growing pigs fed the same human dietary protein source. | | |
| --- | --- | --- |
| Substrate type | Source | Nitrogen |
|  |  | g/kg dry matter |
| Pure protein | Whey protein isolate | 138 |
|  | Whey protein isolate hydrolysate | 130 |
| Ileal digesta | Bovine collagen | 36.4 |
|  | Cheddar | 18.8 |
|  | Chicken | 23.3 |
|  | Eggs | 30.4 |
|  | Feta | 21.1 |
|  | Fish | 24.8 |
|  | Milk | 25.3 |
|  | Cornflakes | 38.5 |
|  | Millet | 33.9 |
|  | Oatmeal | 15.7 |
|  | Rice crackers | 30.2 |
|  | Rye bread | 26.8 |
|  | Sorghum | 24.3 |
|  | Toasted wheat bread | 36.0 |
|  | Wheat bran | 18.5 |
|  | Wheat flour | 13.2 |
|  | Black beans | 24.9 |
|  | Chickpeas | 22.5 |
|  | Kidney beans | 20.0 |
|  | Pigeon peas | 18.7 |
|  | Roasted peanuts | 25.4 |
|  | Mushrooms | 23.8 |
|  | Quorn | 28.0 |
|  | Seaweed | 25.0 |
|  | Spirulina | 33.15 |
|  | Yeast | 23.30 |
|  | Amaranth | 35.7 |
|  | Buckwheat | 16.0 |
|  | Linseed | 23.4 |
|  | Potato | 15.9 |
|  | Potato protein | 26.3 |
|  | | |

**Reference**

1. Hulse, J. H., K. O. Rachie, and L. W. Billingsley (Eds.). 1977. Nutritional standards and methods of evaluation for food legume breeders (IDRC-TS 7e). International Development Research Centre (IDRC), Ottawa, Canada.
2. Hodgkinson SM, Stein HH, de Vries S et al. (2020). Determination of true ileal amino acid digestibility in the growing pig for calculation of digestible indispensable amino acid score (DIAAS). *J. Nutr.* **150**, 2621-2623.
